# Microclimatic effects on alpine plant communities and flower-visitor interactions

**DOI:** 10.1038/s41598-020-58388-7

**Published:** 2020-01-28

**Authors:** Lisa-Maria Ohler, Martin Lechleitner, Robert R. Junker

**Affiliations:** 1 Department of Biosciences, University Salzburg, 5020 Salzburg, Austria; 2 Evolutionary Ecology of Plants, Faculty of Biology, Philipps-University Marburg, 35043 Marburg, Germany

**Keywords:** Biodiversity, Community ecology

## Abstract

High-alpine ecosystems are commonly assumed to be particularly endangered by climate warming. Recent research, however, suggests that the heterogeneous topography of alpine landscapes provide microclimatic niches for alpine plants (i.e. soil temperatures that support the establishment and reproduction of species). Whether the microclimatic heterogeneity also affects diversity or species interactions on higher trophic levels remains unknown. Here we show that variation in mean seasonal soil temperature within an alpine pasture is within the same range as in plots differing in nearly 500 m in elevation. This pronounced heterogeneity of soil temperature among plots affected the spatial distribution of flowering plant species in our study area with a higher plant richness and cover in warmer plots. This increased plant productivity in warmer plots positively affected richness of flower visitor taxa as well as interaction frequency. Additionally, flower-visitor networks were more generalized in plots with higher plant cover. These results suggest that soil temperature directly affects plant diversity and productivity and indirectly affects network stability. The strong effect of heterogeneous soil temperature on plant communities and their interaction partners may also mitigate climate warming impacts by enabling plants to track their suitable temperature niches within a confined area.

## Introduction

Alpine ecosystems are particularly sensitive to climate change as scenarios predict severe warming for high elevations in alpine regions^1^. Therefore, cold adapted alpine plant species are threatened by increased temperatures^2,3^. Recently, considerable work has described and tested possible scenarios for responses of alpine plants to climate change^4,5^. Regional plant diversity in alpine habitats is expected to change as a result of climate warming due to shifts in temperature niches and species’ distributions, which may result in increased competition due to shifts of plants’ distributions to higher elevations and consequently the assembly of new communities^6–8^. Plant species richness and composition affects organisms and processes across trophic levels^9^. For instance, the diversities of plants and their flower visitors have been shown to be particularly related due to insect-specific preferences for certain plant species^10–12^. Therefore, a change in plant abundance and distribution and shifts in phenologies due to rising temperatures potentially have negative effects on other trophic levels, in particular on flower visiting insects^13,14^ and thus impact community structure and put ecosystem functions at risk^15^.

Among the main drivers of (alpine) plant diversity are climatic conditions and biotic interactions^16–19^. However, plant community composition and diversity are not only shaped by average environmental and climatic conditions but also by local micro-abiotic filtering^20^. Specifically, soil temperatures, which in contrast to air temperatures are strongly shaped by the local topography and intake of solar radiation^21,22^, are thought to affect photosynthetic capacity and growth rates of plants^23–25^. Consequently, fine-scale environmental heterogeneity can also shape functional traits as well as community structure of plants^26^. However, the relationship of heterogenous soil temperature (i.e. microclimate), species richness and composition remains understudied^27^ and whether differences in local soil temperature also affect higher trophic levels such as flower visitors remains unknown. Plant–insect interactions are affected by climate warming possibly leading to temporal and spatial mismatches among mutualistic partners^13^. Temperature is the main trigger of flowering phenology for alpine species and local variations in temperature can lead to a shift in flowering phenology^28,29^, which may be particularly relevant in alpine landscapes^30–32^. We hypothesize bottom-up effects of small-scale soil temperature heterogeneity not only on plant communities but also on flower visiting insects and plant-insect interactions. This effect across trophic levels may have implications for the potential of microclimate as buffer for climate change impacts by increasing overall diversity and by reducing possible mismatches in phenologies of plants and insects. To test for these possible direct and indirect effects of soil temperature we recorded mean seasonal soil temperature, plant communities and plant-insect interactions on 30 small-scale (1.5 × 1.5 m) microclimatic plots within 1.25 ha on a topographically heterogenous alpine pasture on the same elevation. We hypothesized that the mean seasonal soil temperature either directly or indirectly affects plant and animal communities as well as their interactions. Based on pre-existing knowledge from the literature and own experience, we more specifically hypothesized that our main effect, the mean seasonal temperature of the plots, directly and positively affects plant cover and plant species richness^18^. We further hypothesized direct positive effects of plant cover and plant richness on insect family richness^11,33^. Lastly, we expected a direct positive effect of plant cover as well as plant and insect species richness on the number of plant-insect interactions and a negative relationship of these variables with complementary specialization *H*_2_’^10,12,19^. To test these hypotheses we conducted a path analysis, which can be useful for investigating complex causal relationships in ecosystems and between different trophic levels^34,35^.

This study is aiming to investigate microclimatic differences in root zone temperature of small-scale plots within a topographically heterogeneous alpine pasture with neglectable differences in elevation, as well as the effect of local soil temperature on plant and flower visitor communities, plant-insect interactions and network specialization.

## Material and Methods

### Field site and mean seasonal soil temperature

Field work was conducted in the mountain range of the Hohe Tauern in the Austrian Alps. The study site (12°49′21″ E 47°07′24″ N) was located on an alpine pasture at an elevation of 2,273 m a.s.l. The study site was confined by mountain ridges in the north and east, which resulted in a shorter daily period of direct solar radiation in the eastern part of the study site than in the western part. The pasture was characterized by a heterogeneous topography forming flat hills, which also introduced variation in the time of solar radiation and also in angle of sunlight. Shortly after snow melt in 2016, we established 30 plots (1.5 × 1.5 m) within an area of 1.25 ha. The location of plots was chosen to represent the variability in aspect of the hills (Supplementary Table 2). The maximum elevational difference between plots was 21.90 m, suggesting that microclimatic heterogeneity results from differences in position, exposition and inclination but not from differences in elevation. The mean seasonal soil temperature of each plot was measured with a temperature logger (DS1921G-F5 Thermochron iButtons, Maxime Integrated Products, Sunnyvale, CA, USA) with a resolution of 0.5 °C. Each plot was equipped with a temperature logger that was wrapped in a plastic bag and buried in the centre of the plot at a soil depth of 3 cm^21^. The soil temperature was measured continuously every 30 minutes from June 24^th^ 2016 to September 06^th^ 2016 (Supplementary Table 2), which covers the full vegetation period at this elevation. In order to relate the microclimatic heterogeneity within the study site to temperature differences along the elevational gradient in the same Alpine region, we selected eight comparable pastures between 1227 and 2634 m a.s.l and recorded the mean seasonal soil temperature in the same way as described above at up to six positions per elevation. Additionally, we used an infrared thermal camera (mobileIR E9, InfraTec GmbH, Dresden, Germany) equipped with a wide-angle lens (13 mm/40.53° × 30.96°) to assess the overall heterogeneity (i.e. standard deviation) in surface temperature of the whole study area. For this we mounted the infrared thermal camera on top of a higher elevated hill in the east of the study area and took an image of the whole study area at noon at the peak of the vegetation season. To investigate the heterogeneity in surface temperature within each of the single study plots we took infrared thermal pictures of every plot seven to nine times a day on four days following a randomized order to account for different weather conditions and then calculated the variations in soil temperature heterogeneity over the course of the day using a quadratic model. Thermal photographs were analysed using the software IRBIS 3plus (mobileIR E9, InfraTec GmbH, Dresden, Germany).

### Vegetation and flower-visitor interactions

Once per week throughout the vegetation season, we recorded the floral abundance of all entomophilous plant species in anthesis per plot (Supplementary Table 3). Floral abundance was defined as the number of floral units (i.e. individual flowers or inflorescences). Thus, the species richness of flowering plants per plot was defined as the total number of flowering entomophilous species per plot throughout the vegetation period. The information on flower abundance per species per plot was also used to compare the community composition of flowering plants between the different plots. Total plant cover per plot in percent was recorded once in the middle of the growing season (Supplementary Table 2). At each plot, flower-visitor interactions were recorded at four events between 07.07.2016 and 18.08.2016 for a five-minute period (Supplementary Table 4). Each sampling day, plots were visited in a randomized order to avoid temporal and spatial biases. The observation of insect visitation was conducted during clear sky conditions. All insects interacting with flowers in anthesis were captured in plastic jars for subsequent determination on family level, if possible, on genus or species level (detailed list of taxa provided in Supplementary Table 5). A statistical analysis on the species level is not meaningful due to the high diversity in arthropod species resulting in only one or few observations per species. From these recordings we received the number insect families as well as the total number of insects interacting with flowers for each plot and additionally calculated the complementary specialization *H*_2_’ of the insect-plant network per plot using the R package *bipartite*^36^. Complementary specialization *H*_2_’ is a network index, which measures the degree of exclusiveness of interactions on the level of the entire network^37^

### Statistical analysis

#### Microclimatic heterogeneity relative to temperature differences along an elevational gradient

In order to relate the microclimatic heterogeneity with the temperature differences along the elevational gradient, we exploited temperature data from two sources. We used our own temperature logger measurements at eight elevations between 1227 and 2636 m a.s.l. (see above) and additionally, we retrieved mean temperature data (1979–2013) from CHELSA (Climatologies at high resolution for the earth’s land surface areas;^38,39^) based on a downscaled high-resolution output of the ERA Interim (European re-analysis;^40^) model for the months June, July, August, September, which corresponds to the period of the year when we used the temperature loggers. We estimated linear regression models to determine the relationship between elevation and temperature in our study region and used this model to evaluate which differences in elevation correspond to the temperature variations among the microclimatic plots. To test whether mean seasonal soil temperature is affected by plot orientation we fitted a linear model and additionally applied an Estimated Marginal Means test carried out with the R package *emmeans* 1.4^41^.

#### Flowering plant species composition

To test whether the plant species composition is affected by the mean seasonal soil temperature on micro-plots, we performed a Constrained Analysis of Principal coordinates (CAP) as implemented in the R package *vegan* 2.4–2^42^ based on the quantitative composition of flowering plant species (floral abundance counts, Bray-Curtis distances) and based on the mean seasonal soil temperature measured on the same plots (Euclidean distances). Furthermore, the Bray-Curtis distances reflecting the dissimilarity in qualitative plant species composition were visualized using an NMDS calculated with the R package *MASS* 7.3–51.1^43^ and temperatures at these plots were added with a thin plate spine surface for interpolating and smoothing the data using the R package *fields* 9.6^44^.

#### Relationship between microclimatic soil temperature on plant and insect richness and their interactions

We applied path analysis to test for the relationships of mean seasonal soil temperature, plant cover, species richness of flowering plants, number of insect families recorded on flowers, number of observed insect interactions with flowers and complementary specialization *H*_2_’ of flower-visitor interaction networks. We fitted the path model according to our hypotheses summarized in the introduction, using default settings of the R package *lavaan* and a maximum likelihood (ML) estimator^45^.

## Results

### Mean seasonal soil temperature

**Figure 1:**
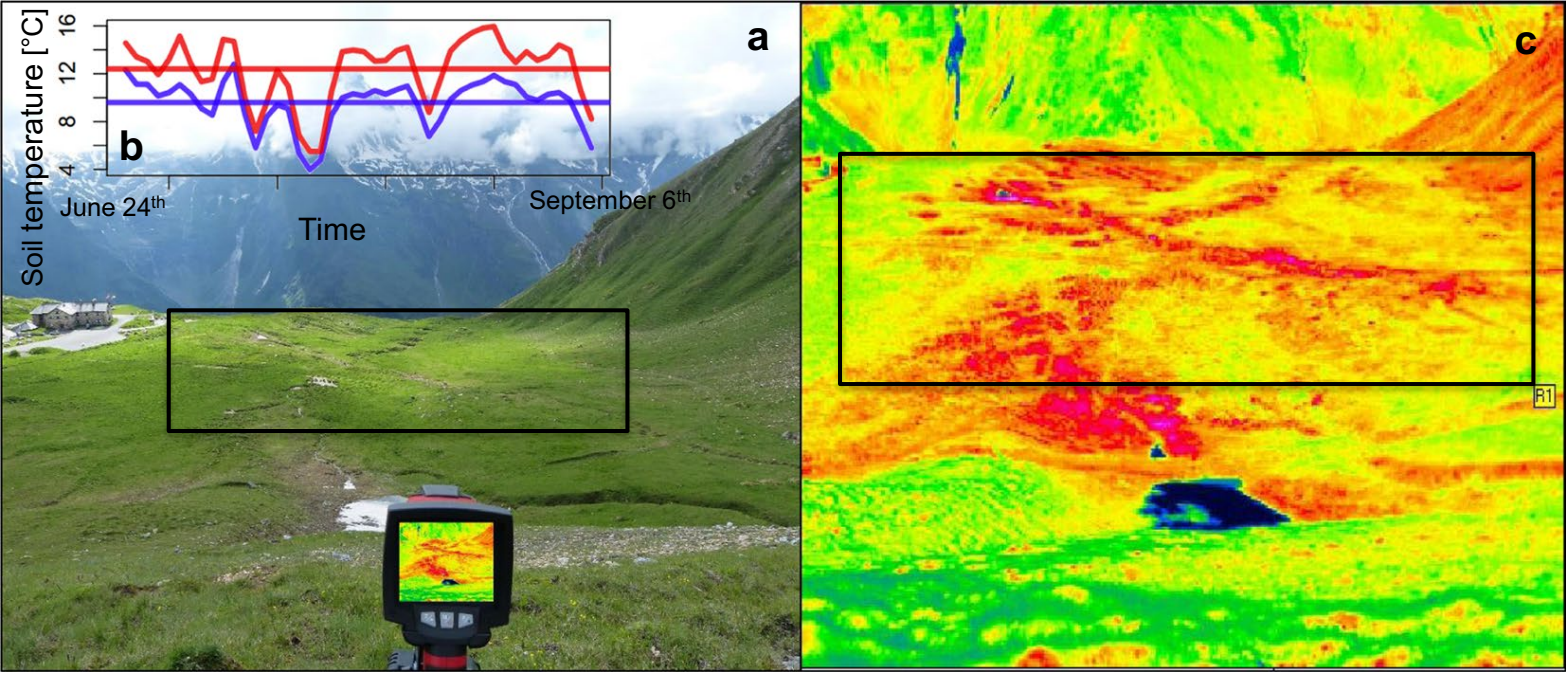
Study site with pronounced microclimatic heterogeneity. (**a**) Study site, a high alpine pasture with heterogenous topography located at 2,273 m a.s.l. in the mountain range of the Hohe Tauern in the Austrian Alps (12°49′21″ E 47°07′24″ N). The *n* = 30 plots were distributed within the black frame. (**b**) Seasonal course of soil temperature of the vegetative season in 2016 of the warmest (red) and the coldest plot (blue) measured with temperature loggers. The horizontal lines indicate the mean seasonal soil temperatures of the warmest (upper line, red) and coldest plot (lower line, blue). (**c**) False color image of the study site taken with an infrared thermal camera showing the microclimatic heterogeneity in surface temperature at noon. Each pixel represents the surface temperature of a given position. Black to blue pixels represent cold surface temperatures (e.g. the snowfield in the foreground), warmer colours are represented by a gradient from green over yellow to red and pink. The *n* = 30 plots were distributed within the black frame.

**Figure 2:**
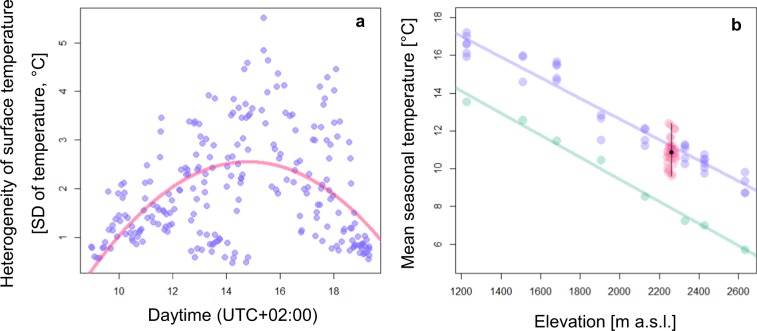
Diurnal course of surface temperature heterogeneity within plots and mean seasonal temperature across plots and spatial scales. (**a**) Microclimatic heterogeneity (i.e. standard deviation in surface temperature within plots) of all plots across the study site recorded by an infrared thermal camera following a diurnal course calculated with a quadratic model (red line). *n* = 30 plots (2.25 m²) were photographed up to nine times at different times of the day between 0900 and 1930 (UTC+02:00). (**b**) Mean seasonal temperature measurements of different sources and scales within the study area. Mean seasonal soil temperature of *n* = 30 plots (red points) on an alpine pasture located at 2,273 m a.s.l. in the mountain range of the Hohe Tauern in the Austrian Alps. Mean seasonal mean soil temperature and standard deviation are given in black. Seasonal mean soil temperature at eight elevations between 1227 and 2634 m a.s.l. in the same area measured with temperature loggers (blue points), regression line is given as blue line. Per elevation, three loggers were used. Air temperature data (1979–2013) retrieved from CHELSA climate database for the same eight elevations (green), regression line is given as green line.

The mean seasonal soil temperature of our study plots measured with temperature loggers in 3 cm belowground ranged between 9.60 °C and 12.40 °C (mean ± SD = 10.88 ± 0.75 °C). The largest difference in mean seasonal soil temperature between two study plots was therefore 2.8 °C (Fig. 1). Microclimatic heterogeneity of local surface temperature within plots as recorded by an infrared thermal camera was subjected to strong diurnal variations (quadratic model: *F*_2, 239_ = 39.73, *p* < 0.001, *r*^2^ = 0.24, Fig. 2a). Surface temperature heterogeneity peaked between 1400 and 1500 (UTC + 02:00) when temperature was distributed most heterogeneously across the plots indicating that solar radiation is the source of microclimatic heterogeneity in our system. To support this finding, we tested the effect of plot orientation (i.e. aspect; Supplementary Table 2) on mean seasonal soil temperature (linear model: *F*_4,25_ = 5.20, *p* < 0.01) and found that temperature differed most between north and south oriented plots with the southern plots being significantly warmer (Estimated Marginal Means: *t*_25_ = −3.39, *p* = 0.01). Additionally, we tested for spatial autocorrelation of mean seasonal soil temperature along the spatial distribution within the study area, using redundancy analysis, which summarizes linear relationships between response variables that are explained by a set of explanatory variables (here the geographic location of the plots) by allowing regression of multiple variables. This analysis indicated no significant relationship of mean seasonal soil temperature with the spatial position of the plots (*F*_2,27_ = 2.82, *p* = 0.09).

Mean seasonal temperature of other eight pastures between 1227 and 2636 m a.s.l. clearly decreased with increasing elevation both based on our own measurements using temperature loggers buried in soil (Pearson’s product-moment correlation: *t*_34_ = −24.84, *p* < 0.001, *r*^2^ = 0.95, slope of regression: −0.0055) and based on the data provided by CHELSA (*t*_6_ = −24.03, *p* < 0.001, *r*^2^ = 0.99, slope of regression: −0.0058, Fig. 2b)^38,39^. The mean of the mean seasonal soil temperatures (10.88 °C) measured on the 30 plots at our microclimatic heterogeneous pasture was similar to the temperature as predicted by the regression model (Fig. 2b). However, variation between the plots was pronounced (95% confidence interval: 9.67 – 12.33). This range of temperature corresponds to temperature differences expected in elevations differing in 484 or 474 m following the regression models based on our temperature logger or CHELSA data, respectively (Fig. 2b).

### Flowering plants, insects and their interactions

The total plant cover within the plots ranged between 70% and 100% (mean ± SD = 90.93 ± 7.24). A total of *n* = 59 entomophilous forb species were in anthesis during the observation period. The number of flowering entomophilous forbs (species richness) varied from *n* = 10 to *n = *23 species per plot (mean ± SD = 16.5 ± 3.41). The number of insect families per plot interacting with flowers (insect family richness) varied from *n* = 3 to *n* = 20 (mean ± SD = 8.77 ± 3.84). The sum of insect interactions with flowers per plot was between *n* = 5 and *n* = 80 (mean ± SD = 32.60 ± 18.22). Interactions between flowers and their visitors were on average rather generalized (network specialization index *H*_2_’: mean ± SD = 0.37 ± 0.20).

### Relationship between heterogeneous soil temperature, species composition and richness, and flower-visitor interactions

**Figure 3:**
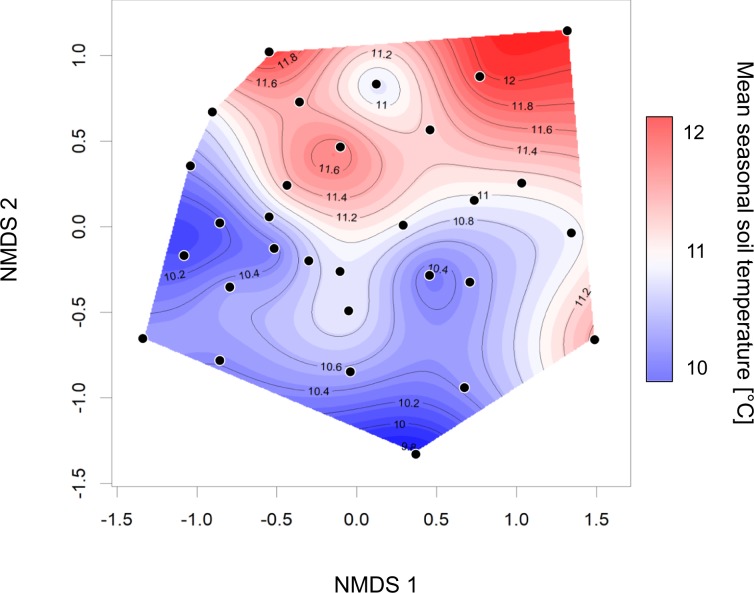
Similarity of study plots in flowering plant species composition in relation to seasonal mean soil temperature. Non-metric multi-dimensional scaling (NMDS) of the dissimilarity (Bray-Curtis) in plant community composition (i.e. beta-diversity) between *n *= 30 investigated 1.5 ×1.5 m plots on an alpine pasture located at 2,273 m a.s.l. in the mountain range of the Hohe Tauern in the Austrian Alps. The distance between the points is a measure for the dissimilarity in the community composition (i.e. the further two points are apart the more they differ). Information on mean seasonal soil temperature of each plot was added by interpolating and smoothing the data. Thus, position of plots in the ordination is determined by plant species composition, temperature information was added in a second step. Red background colors indicate warmer mean seasonal soil temperatures and blue colors resemble colder temperatures measured in each plot. Plant species that specifically responded to mean seasonal soil temperatures are shown in Supplementary Figure 1.

Plant species composition varied between plots and responded to variation in mean seasonal soil temperature, which means that plots with similar temperature were also similar in plant species composition (permutation test for CAP: *F*_1,28_ = 1.76, *p* < 0.01, Fig. 3). Plant species that specifically contributed most to the differences in community composition by significantly responding to mean seasonal soil temperatures are shown in Supplementary Figure 1.

**Figure 4:**
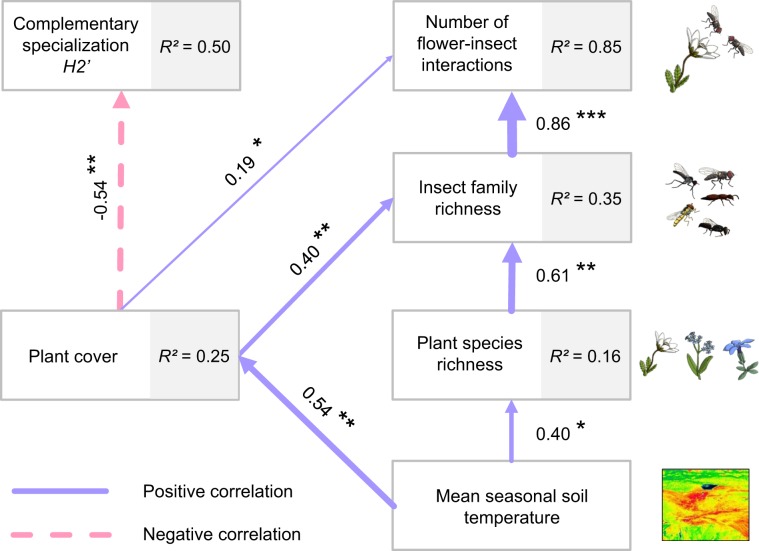
Path analysis of mean seasonal soil temperature and parameters characterizing plant and animal communities as well as their interactions. Path analysis explaining the causal relationships between mean seasonal soil temperature, plant cover, species richness of flowering plants, number of insect families recorded on flowers, number of observed insect interactions with flowers and complementary specialization *H*_2_’ recorded on *n *= 30 plots (2.25 m²) on a topographically heterogenous alpine pasture located at 2,273 m a.s.l. in the mountain range of the Hohe Tauern in the Austrian Alps (12°49′21″ E 47°07′24″ N). Arrows indicate significant relationships between the tested parameters (blue: positive effect, pink: negative effect). Numbers next to the arrows indicate standardized path coefficients, which are also resembled in the arrow width and asterisks mark their significance (*, *p* < 0.5: **, *p* < 0.01; and ***, *p* < 0.001). Numbers next to the endogenous variables indicate the proportion of their total explained variance (R²).

We tested the effect of local mean seasonal soil temperature on plant communities and flower-visitor interactions using path analysis (Fig. 4), which largely supported our hypotheses. Coinciding with our hypotheses that mean seasonal soil temperature directly or indirectly affects all other variables we found that it positively and directly affected plant cover (path analysis: standardized path coefficient *β* = 0.54, *p* < 0.01) and the number of flowering plant species of plots (*β* = 0.40, *p* = 0.04). Further we hypothesized a direct positive effect of plants on insect richness and found that the number of insect families had a positive relationship with both plant cover (*β* = 0.40, *p* < 0.01) and plant species richness (*β* = 0.61, *p* < 0.01). Finally, we hypothesized that plant cover as well as plant and insect richness positively affect the number of plant-insect interactions and negatively affect complementary specialization *H*_2_’, leading to a more generalized network. The number of insect-flower interactions showed a positive relationship with plant cover (*β* = 0.19, *p* = 0.02) and insect family richness (*β* = 0.86, *p* < 0.001), but was unaffected by the number of plant species (*β* = 0.02, *p* = 0.87). Complementary specialization of networks *H*_2_’ was unaffected by plant (*β* = −0.01, *p *= 0.92) and insect richness (*β *= −0.34, *p *= 0.20). However, we detected a negative relation between total plant cover and *H*_2_’ (*β *= −0.54, *p* <0.01). Apart from the results described above, no other relationship between the tested variables shown in Fig. 4 was significant (Supplementary Table 1).

## Discussion

Microclimatic heterogeneity is particularly pronounced in alpine environments^21^ and has been suggested to affect the diversity and composition of plant communities^18^. However, whether microclimatic effects also affect higher trophic levels, such as flower visitors, remains unknown. In our study area we detected strong microclimatic heterogeneity with differences in mean seasonal soil temperature of 2.8 °C between our study plots (Fig. 1). The magnitude of temperature heterogeneity across plots is especially pronounced during midday, when the highest soil temperatures are reached (Fig. 2a). The aspect of the plots, which determines the intake of solar irradiance, was shown to be the main cause of microclimatic heterogeneity with the highest difference between colder north and warmer south facing slopes. This finding further suggests that these microclimatic temperature trends may be consistent across years. Other factors such as air temperature, wind speed, surface albedo, microtopography, substrate type, and evapotranspiration may additionally increase temperature variability^21,25,46^.

This temperature difference between plots that were located in close vicinity and on the same elevation corresponds to the temperature difference as measured between sites that differ in nearly 500 meters in elevation (Fig. 2b), which is also in agreement with an average decrease in air temperature of 0.6 °C per 100 meters upslope^47^. The extent of temperature variation provided by microhabitats due to local small-scale topography found in this study corresponds to the most likely warming scenarios for the next 100 years of 3 °C as reported in the Intergovernmental Panel on Climate Change (IPCC) projections. Therefore, the pronounced microclimatic heterogeneity suggests that microclimatic habitats are capable of providing sufficient heterogeneity to buffer climate change impact on alpine plants^46,48,49^ and on higher trophic levels dependent on plant resources. However, the overall distribution of microclimatic soil temperatures will shift towards warmer temperatures in the predicted warming scenarios and a small percentage of micro-habitats will be lost^49^. Nevertheless, microclimatic heterogeneity may reduce the necessity of upward migrations of species as a response to climate warming. In conclusion, alpine plants and their consumers can experience the same climatic conditions in climate refuges within a few meters distance on the same elevational level. Therefore, migration of a few meters may be sufficient to track their suitable temperature niche.

Microclimatic heterogeneity may thus have the capability of buffering climate warming impacts on alpine plants and their interactions with flower visitors by reducing possible mismatches in phenologies *via* desynchronized flowering at different temperatures and by increasing overall diversity, leading to more stable systems as suggested by our results. In line with this notion, plant communities responded to increased temperatures with higher cover and higher species richness in flowering plants (Fig. 4). Next to species numbers, microclimate also affected plant species composition with different species assemblages on plots with different mean seasonal soil temperatures (Fig. 3). These effects of microclimate on the alpha- and beta-diversity of plant communities lead to a higher richness of insects consuming floral resources on warmer plots. No direct effect of soil temperature on insect visitors was evident. Therefore, diversity of higher trophic levels is suggested to be indirectly bottom-up controlled by the effect of microclimatic soil temperatures on plants which in turn directly affected flower visitors. In addition to the direct microclimatic effects on plant communities and the indirect effects on insects, microclimatic heterogeneity also affects the structure of interaction networks with more generalized interactions on warmer plots than on colder ones (Fig. 4).

In concordance to earlier findings, we found a higher vegetation cover on warmer plots compared to colder plots, which may be attributed to a positive relationship between plant productivity and temperature demonstrated in several studies^22,50,51^. Higher temperatures further affected species richness of insect pollinated plants with a larger number of species on warmer plots (see also^33^). Community composition of entomophilous flowering plants also responded to different soil temperatures with more similar species assemblages in more similar soil temperatures. Thus, microclimatic heterogeneity on a local scale (e.g. in our spatially restricted study area) may offer more temperature niches that environmentally filter different assemblages of plant species^52–56^, which leads to a larger number of plants that are able to establish in a given area^6,18,20,57^. Thus, alpha-diversity of plants in an alpine habitat is facilitated by a high small-scale beta-diversity, i.e. by changing species assemblages due to spatial variations in soil temperature.

Additional to the finding that the heterogeneity in soil temperature affects plant species assemblages, we demonstrated that also higher trophic levels (e.g. flower visiting insects) and interactions between trophic levels respond to microclimate. Enthomophilous plants depend on insects for reproductive success and, in turn, flower visiting insects depend on flowering plants for resources suggesting a tight dependency between these trophic levels, potentially even on smaller scales. Flower visiting insects are believed not to be directly influenced by soil temperature because the activity of flower visitors is mainly affected by larger scale weather conditions, such as air temperature, solar radiation and wind^58,59^. Correspondingly, we found no direct effect of local soil temperature on the richness of flower visitors. However, we found an indirect bottom-up effect of microclimate on the number of flower visiting insect families, which increased with higher flowering plant species richness found at warmer plots. Species richness of insect communities has been shown to be closely related to floral diversity (i.e. resource diversity^10,60^. This finding may be explained by the fact that different species of flower visitors exhibit a specialization to specific floral traits^11^. Communities with a larger number of plant species are assumed to offer more niches that can be occupied by a higher number of flower visiting insect species specialized to specific flower traits^12^. Furthermore, a higher number of insect families led to more insect-flower interactions in warmer plots, while the number of interactions per taxon was unaffected by taxon richness per plot. Therefore, the higher number of interactions results from an additive effect of more interacting taxa instead of more frequent interactions per taxon. Interestingly, not only was the richness of plant and animals directly or indirectly affected by microclimate, but interactions between these trophic levels also varied with mean seasonal soil temperature. Interaction networks were more generalized (i.e. lower *H*_2_’) on warmer plots that also featured a higher plant cover. While we have no clear mechanistic explanation for this finding, these results are however suggesting that warmer plots favour productivity (higher plant cover)^22,51^ and the stability of the plant and pollinator communities (lower *H*_2_’)^61^. The relationships between plant diversity and productivity^62^ as well as between plant diversity and community stability^61^ are well established. In contrast, the relationship between productivity and community stability has been suggested, only^63^. Our results therefore support the notion that more productive systems may also be more stable – mediated by higher mean seasonal soil temperature – even on a small scale, and therefore may stimulate more research in this direction in the future.

This pronounced small-scale difference in soil temperature affected the spatial distribution of plant cover, richness of flowering plant species and plant species composition. These microclimatic effects on plant communities also affected richness of flower visiting insects as well as the frequency and specialization of plant-insect interactions suggesting an indirect effect of microclimate also on higher trophic levels.

## Supplementary information

Supplementary Information.

Supplementary Dataset 1.

Supplementary Dataset 2.

Supplementary Dataset 3.

Supplementary Dataset 4.

Supplementary Dataset 5.

## Data Availability

The data supporting the results of this study can be found in Supplementary Table 2–5.
