# Supplementary material for: Microclimatic effects on alpine plant communities and flower-visitor interactions
Source: Sci Rep. 2020 Jan 28;10:1366. doi: 10.1038/s41598-020-58388-7 (PMC6987155; doi:10.1038/s41598-020-58388-7)
Supplement: Supplementary file 1 — Supplementary Information. [file 41598_2020_58388_MOESM1_ESM.pdf]

Microclimatic effects on alpine plant communities and flower-visitor interactions

Lisa-Maria Ohler, Martin Lechleitner, Robert R. Junker

Supplementary Information 1

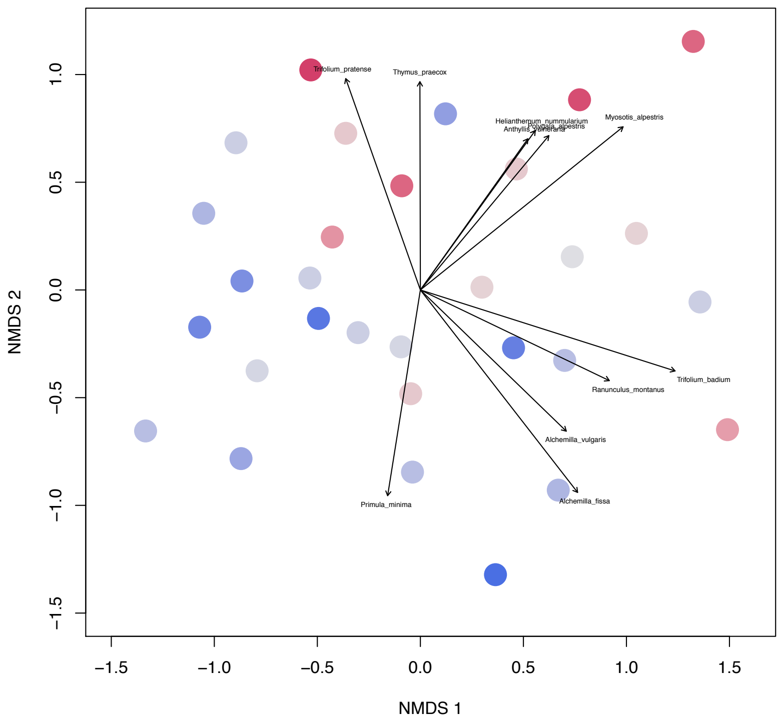

Supplementary Fig. 1 Similarity of study plots in flowering plant species composition in relation to seasonal mean soil temperature. Non-metric multi-dimensional scaling (NMDS) of the dissimilarity (Bray-Curtis) in plant community composition (i.e. beta-diversity) between $n = 30$ investigated 1.5 x 1.5 m plots on an alpine pasture located at 2,273 m a.s.l. in the mountain range of the Hohe Tauern in the Austrian Alps. The distance between the points is a measure for the dissimilarity in the community composition (i.e. the further two points are apart the more they differ). The color of the points is reflecting the mean seasonal soil temperature per plot; pink colors indicate warmer mean seasonal soil temperatures and blue colors indicate colder temperatures. Thus, position of plots in the ordination is determined by plant species composition and plant species that specifically responded to mean seasonal soil temperatures ($p < 0.05$) are shown as vector arrows.
